# Mindful attention awareness, creative trait motivation, cognitive flexibility, and achievement goal orientation among medical students: a profile analysis and mediation analysis

**DOI:** 10.3389/fpsyg.2026.1838386

**Published:** 2026-06-24

**Authors:** Qihe Zhong, Yiwen Zhou, Zhengkuan Huang

**Affiliations:** School of Medical Imaging, North Sichuan Medical College, Nanchong, China

**Keywords:** achievement goal orientation, cognitive flexibility, creative trait motivation, medical students, mindful attention awareness

## Abstract

**Objective:**

This study examined the associations among mindful attention awareness, cognitive flexibility, creative trait motivation, and achievement goal orientation in medical students by employing two complementary analytical strategies: a variable-centered approach and a person-centered approach.

**Methods:**

A cross-sectional survey design was adopted. Between December 2025 and February 2026, 460 participants were recruited via convenience sampling from three medical schools in Sichuan Province, China. The assessment instruments comprised the Mindful Attention Awareness Scale, the Creative Trait Motivation Scale, the Cognitive Flexibility Scale, and the Achievement Goal Orientation Scale.

**Results:**

Mindful attention awareness was positively correlated with cognitive flexibility (*r* = 0.322, *p* < 0.001), creative trait motivation (*r* = 0.226, *p* < 0.001), and achievement goal orientation (*r* = 0.143, *p* < 0.01). In the cross-sectional sequential mediation model, after cognitive flexibility and creative trait motivation were simultaneously entered, the direct association between mindful attention awareness and achievement goal orientation was no longer significant (*B* = −0.014, 95% CI = [−0.057, 0.029]), whereas the total indirect association was significant (*B* = 0.075, 95% CI = [0.038, 0.113]). The indirect associations through cognitive flexibility (*B* = 0.015, 95% CI = [0.005, 0.033]), creative trait motivation (*B* = 0.043, 95% CI = [0.013, 0.077]), and their sequential pathway (*B* = 0.016, 95% CI = [0.005, 0.029]) were all significant. Latent profile analysis supported a three-profile solution (Entropy = 0.961), identifying low, high, and moderate mindful attention awareness groups, accounting for 17.64, 43.38, and 38.98% of the sample, respectively. The three profiles differed significantly in cognitive flexibility, creative trait motivation, and achievement goal orientation.

**Conclusion:**

The findings suggest that cognitive flexibility and creative trait motivation may be statistically involved in theory-informed indirect pathways between mindful attention awareness and academic goal-related functioning.

## 1 Introduction

It is well established that medical students are chronically exposed to elevated levels of academic workload, emotional labor, and evaluative pressure (Santiago et al., 2024). This educational ecology places heightened demands on their attentional regulation, emotion management, and sustained intrinsic motivation for academic pursuits (Liang, 2024; Li and Gu, 2025). Consequently, a substantial body of scholarship has investigated the key psychological factors influencing positive learning dispositions and adaptive developmental mechanisms among medical students (Li et al., 2025; Zhang et al., 2022). Among these factors, academic motivation and goal orientation not only shape the quality of learning engagement and knowledge construction (Usán et al., 2019) but also exert a far-reaching influence on future career development trajectories and clinical competence (Ross et al., 2022). Achievement goal orientation refers to the relatively stable motivational tendency through which students define competence, interpret success and failure, and regulate achievement-related behavior in learning contexts (Cheng, 2022; Butera et al., 2024). In achievement goal theory, competence can be pursued and evaluated in different ways. The present study adopted a three-dimensional framework of achievement goal orientation, including mastery goals, performance-approach goals, and performance-avoidance goals (Schwinger and Stiensmeier-Pelster, 2011). Mastery goals emphasize learning, understanding, self-improvement, and competence development (Poortvliet and Giebels, 2012); performance-approach goals emphasize demonstrating competence and outperforming others (Midgley et al., 2001); and performance-avoidance goals emphasize avoiding failure, negative evaluation, or appearing incompetent (Darnon et al., 2007). Thus, achievement goal orientation in this study denotes students’ overall goal-related motivational tendencies in academic achievement contexts rather than a single homogeneous construct. Prior research has demonstrated significant associations between achievement goal orientation and medical students’ learning strategy selection, professional burnout, and clinical reasoning ability (Tekes and Tekin, 2025). Specifically, medical students with higher mastery goal orientation tend to adopt deep learning strategies and exhibit stronger autonomous learning capacity and academic resilience (Orozco-Jiménez et al., 2026), whereas those with higher performance-avoidance goal orientation are more susceptible to surface-level learning and anxiety. Nevertheless, previous studies have paid relatively limited attention to psychological factors that are associated with achievement goal orientation. In particular, identifying which psychological resources and cognitive characteristics are associated with stronger or more adaptive patterns of goal orientation holds considerable theoretical value and practical significance for optimizing medical education strategies and enhancing the quality of talent cultivation.

Although medical students are often broadly categorized as undergraduates, their educational context differs substantially from that of general undergraduate students. Compared with students in many non-medical majors, medical students are typically exposed to a longer training trajectory, denser curricular requirements, more frequent high-stakes assessments, and earlier contact with professional responsibilities (Dyrbye and Shanafelt, 2016). Their learning is not limited to the acquisition of disciplinary knowledge but also involves the continuous integration of biomedical knowledge, clinical reasoning, communication skills, ethical judgment, and professional identity formation (Lindsley et al., 2024; Kim et al., 2024). In particular, clinical clerkships and practicum experiences require medical students to confront uncertain patient conditions, emotionally demanding interpersonal interactions, and the potential consequences of professional errors (Weurlander et al., 2019). Therefore, the psychological resources that support attentional regulation, flexible cognition, intrinsic exploration, and adaptive goal pursuit may be especially important in this population.

These characteristics distinguish medical students from general undergraduates in relation to the core variables examined in the present study. First, mindful attention awareness may be particularly relevant for medical students because they must maintain sustained present-centered attention and awareness of ongoing experience under conditions of academic overload, clinical uncertainty, and emotional stress (Chmielewski et al., 2021; da Silva et al., 2023). Second, cognitive flexibility is central to medical learning because clinical reasoning often requires students to shift among multiple hypotheses, integrate heterogeneous information, and adjust problem representations as new evidence emerges (Custers, 2013; Durning et al., 2016). Third, creative trait motivation is increasingly important in medical education, where students are expected not only to reproduce established knowledge but also to engage in research training, interdisciplinary problem solving, and innovative thinking in response to complex health-care challenges. Finally, achievement goal orientation among medical students has implications beyond academic performance; it may influence deep learning, clinical competence development, professional commitment, and future patient care (Ross et al., 2022). For these reasons, findings derived from general undergraduate samples may not be directly generalizable to medical students, making it necessary to investigate these mechanisms specifically within the medical education context.

In the present study, mindful attention awareness refers specifically to the construct assessed by the Mindful Attention Awareness Scale, namely the tendency to attend to and be aware of present-moment experiences in daily life. The MAAS primarily captures attentional awareness and reduced automaticity in ongoing activities. Therefore, the term ‘mindful attention awareness’ is used throughout this manuscript to refer to present-centered attention and awareness rather than to multidimensional mindfulness as a whole.

Cognitive flexibility reflects an individual’s capacity to adaptively adjust cognitive strategies, shift thinking perspectives, and reconstruct problem representations when confronted with changing environmental conditions or problem contexts (Laureiro-Martínez and Brusoni, 2018; Cheng and Cheung, 2005). Previous research has found that cognitive flexibility is closely associated with clinical reasoning quality, diagnostic accuracy, and interdisciplinary knowledge integration (Durning et al., 2016; Xiong et al., 2025); however, the relationship between mindful attention awareness and cognitive flexibility among medical students has received scant attention. Specifically, through heightened present-centered attentional awareness and reduced automatic responding, medical students may become more conscious of their ongoing cognitive processes and task engagement (Chems-Maarif et al., 2025), thereby attenuating cognitive rigidity and ruminative tendencies and freeing attentional resources that support cognitive flexibility (Solhaug et al., 2016). Subsequently, enhanced cognitive flexibility enables medical students to examine the significance of academic tasks from multiple perspectives (Rudnik et al., 2025) and to reinterpret difficulties and failures as opportunities for learning and growth, thereby strengthening achievement goal orientation. Accordingly, cognitive flexibility may represent a theory-relevant cognitive correlate through which mindful attention awareness is statistically linked with achievement goal orientation.

Creative trait motivation denotes a stable dispositional tendency to engage in creative activities and holds particular significance within the context of medical education (Taylor and Kaufman, 2021). This is primarily because medical learning demands not only the memorization and repetition of knowledge (Price et al., 2025) but also creative thinking about clinical problems, the integration of multi-source information, and the generation of innovative solutions (van Velzen et al., 2024; Liu et al., 2023). Previous studies have demonstrated a positive association between mindfulness and creativity among university students (Khan and Abbas, 2022; Yao et al., 2024). Mindful attention awareness, by heightening present-centered attention to internal experience and to task-relevant external information, may provide favorable conditions for the activation and sustained engagement of creative thinking. More importantly, heightened attentional awareness and reduced automaticity may facilitate disengagement from habitual cognitive patterns and increase sensitivity to novel task-related information (Tempone-Wiltshire and Matthews, 2025), thereby stimulating stronger exploratory motivation and creative interest. In turn, students with stronger creative motivation are more inclined to set goals centered on learning and growth in academic contexts and to demonstrate higher levels of learning engagement and persistence (Peng et al., 2013; Burleson, 2005). Thus, the experiential openness and internal sensitivity engendered by mindful attention awareness first activate an individual’s interest in and propensity for engagement in creative activities, and this creative motivational orientation may be associated with stronger mastery- and growth-related achievement goal tendencies.

According to the dual-pathway model of creativity (Nijstad et al., 2010), cognitive flexibility enhances creative performance by facilitating remote associations and multi-perspective exploration (Li et al., 2026). Extending this reasoning, cognitive flexibility and creative trait motivation may exhibit a progressive functional linkage, jointly influencing the relationship between mindful attention awareness and achievement goal orientation. Specifically, from a theory-informed perspective, mindful attention awareness may be positively associated with cognitive flexibility, possibly because greater present-centered awareness is linked to reduced automaticity and more flexible allocation of attention (Musood and Kaur, 2025). Higher cognitive flexibility may then be associated with a greater tendency to appraise learning tasks and problem contexts from more diversified perspectives, thereby generating a broader possibility space and a richer set of associative pathways at the cognitive level. This cognitive openness and flexibility may provide a theoretically plausible basis for stronger creative trait motivation (Shao et al., 2018). In other words, when individuals possess a higher level of cognitive flexibility, they are more likely to experience novelty and a sense of competence during divergent thinking and problem-solving processes, which in turn strengthens their intrinsic motivation to engage in creative activities.

Analysis of psychological mechanisms in medical education has traditionally relied on variable-centered approaches, which estimate average associations among constructs under the implicit assumption that the sample is relatively homogeneous with respect to the focal psychological processes (Tempelaar and Niculescu, 2026; Chen et al., 2025). Although such approaches are useful for clarifying general patterns of association, they may obscure meaningful heterogeneity among students who differ in their attentional regulation, emotional reactivity, learning experiences, and adaptive resources. This issue is particularly relevant in medical education, where students are exposed to diverse developmental contexts, including differences in academic stage, clinical practicum experience, examination pressure, research involvement, and workload intensity (Dornan et al., 2006). These heterogeneous educational and developmental experiences may lead to distinct configurations of mindful attention awareness within the same medical student population.

From a theoretical perspective, mindful attention awareness is not merely a uniform psychological attribute distributed evenly across individuals. Rather, it reflects a self-regulatory capacity involving present-centered attention, awareness of ongoing experience, and reduced automaticity in responding to internal and external events (Vago and David, 2012). Individual differences in attentional control, stress exposure, emotion regulation capacity, and learning context may therefore produce qualitatively different patterns of mindful attention awareness (Prakash et al., 2015; Tang and Braver, 2020). Medical students with lower mindful attention awareness may be more likely to operate in an automatic or reactive mode when facing academic demands, whereas those with higher mindful attention awareness may be better able to maintain conscious engagement with present tasks and monitor their cognitive and emotional responses. Students at intermediate levels may show partial or situationally dependent mindful functioning. Thus, there are theoretical reasons to expect that mindful attention awareness among medical students may be characterized by distinct latent subgroups rather than by a purely homogeneous continuum.

Empirically, prior person-centered research has increasingly demonstrated that mindfulness-related characteristics can form distinguishable latent profiles. For example, latent profile and related person-centered studies have identified subgroups with low, moderate, and high mindfulness-related functioning in general populations and intervention samples (Strohmaier and Medvedev, 2025; Bravo et al., 2016). These findings suggest that mindfulness is not only associated with average-level differences in psychological outcomes but may also appear in qualitatively distinct patterns across individuals. In addition, person-centered analyses have shown that different mindfulness profiles are often associated with different levels of psychological adjustment, emotional functioning, and adaptive behavior (Bravo et al., 2018). Although these studies provide initial support for the heterogeneity of mindfulness-related characteristics, relatively little is known about whether such latent profiles exist among medical students, a population facing substantial academic, clinical, and emotional demands. Therefore, applying latent profile analysis to mindful attention awareness in medical students can extend previous mindfulness profile research and provide a more precise understanding of psychological heterogeneity in this specific educational context.

Moreover, there are strong theoretical reasons to expect that different mindful attention awareness profiles would differ in cognitive flexibility, creative trait motivation, and achievement goal orientation. Mindful attention awareness involves the capacity to notice ongoing experiences without being dominated by automatic reactions (Raffone et al., 2010). This capacity may facilitate cognitive flexibility by helping students disengage from rigid interpretations and shift perspectives when encountering complex academic or clinical problems. Students with higher mindful attention awareness are therefore more likely to reconstruct problem representations, consider alternative solutions, and flexibly adjust learning strategies. In contrast, students with lower mindful attention awareness may be more vulnerable to cognitive fixation and habitual responses, which may constrain flexible thinking.

Mindful attention awareness may also distinguish students in terms of creative trait motivation. By enhancing present-centered attention to internal experience and to external information, mindful awareness may allow students to notice curiosity, novelty, and exploratory interests more fully (Tan et al., 2023). This present-centered and less automatic mode of processing may weaken habitual thought patterns and facilitate engagement with creative activities (Verhaeghen et al., 2017). Accordingly, students characterized by higher mindful attention awareness may display stronger intrinsic interest in creative exploration, whereas those with lower mindful attention awareness may show weaker creative engagement because of attentional dispersion, automaticity, or reduced sensitivity to internally generated interests.

Finally, different mindful attention awareness profiles may be associated with different levels of achievement goal orientation. Achievement goal orientation depends not only on academic ability but also on how students interpret challenge, failure, competence development, and learning value. Students with higher mindful attention awareness may be more capable of attending to academic difficulties with reduced automatic reactivity and dispersion, thereby construing challenges as opportunities for growth and competence development. Such an adaptive appraisal process is theoretically consistent with stronger mastery- and growth-oriented goal tendencies. Conversely, students with lower mindful attention awareness may be more likely to respond automatically to academic pressure and evaluation, which may weaken adaptive achievement goal orientation. Therefore, examining whether latent profiles of mindful attention awareness differ in these cognitive, motivational, and goal-related outcomes is theoretically meaningful and empirically necessary.

Based on the foregoing theoretical and empirical considerations, the following hypotheses were proposed:

*H1*: Mindful attention awareness is positively associated with achievement goal orientation among medical students.

*H2*: Cognitive flexibility is statistically involved in the indirect association between mindful attention awareness and achievement goal orientation.

*H3*: Creative trait motivation is statistically involved in the indirect association between mindful attention awareness and achievement goal orientation.

*H4*: Cognitive flexibility and creative trait motivation jointly constitute a theory-informed sequential indirect pathway linking mindful attention awareness with achievement goal orientation.

*H5*: Medical students can be classified into distinct latent profiles based on their mindful attention awareness.

*H6*: The identified latent profiles of mindful attention awareness differ significantly in cognitive flexibility, creative trait motivation, and achievement goal orientation.

## 2 Methods

### 2.1 Study design

This study employed a cross-sectional survey design. Prior to implementation, the study was reviewed and approved by the Medical Ethics Committee of North Sichuan Medical College (No.: 2025038). Questionnaire data were collected and managed using anonymous coding to ensure data confidentiality. Before participation, students were informed of the study purpose, procedures, voluntary nature of participation, anonymity, confidentiality, potential risks and benefits, and their right to withdraw at any time without penalty. Written/electronic informed consent was obtained from all participants before data collection. Participants could withdraw from the study at any time before submitting the questionnaire. For online questionnaires, participants could withdraw by closing the survey webpage; for paper-based questionnaires, participants could withdraw by returning a blank questionnaire or asking the researcher not to collect their questionnaire. Data from participants who withdrew before submission were not retained or analyzed. Because the survey was anonymous after submission and no personally identifying information was collected, individual responses could not be identified or withdrawn after submission. This was clearly stated in the informed consent form. All data were collected anonymously and stored in password-protected electronic files accessible only to authorized members of the research team. The dataset used for analysis contained no personally identifiable information and was used only for research purposes. No monetary compensation, course credit, gifts, or other incentives were provided for participation.

Participant recruitment. This study used a two-stage non-probability sampling procedure and was conducted between December 2025 and February 2026 across three medical schools in Sichuan Province, China. In the first stage, three medical schools in Sichuan Province, China, were selected by convenience sampling based on institutional accessibility, permission to conduct the survey, and feasibility of participant recruitment. In the second stage, within each participating school, classes were used as sampling units. All students in the selected classes who met the inclusion criteria were invited to participate in the survey. Thus, the sampling procedure combined convenience selection of schools with random selection of classes within the participating schools. Researchers contacted class advisors at each institution and obtained permission for their assistance. Survey questionnaire links and corresponding QR codes were distributed to students in the selected classes via a professional data collection platform. The cover page of the electronic questionnaire informed participants of the study objectives, content, voluntary nature of participation, anonymity, confidentiality, and potential benefits and risks. Participants could proceed to complete the questionnaire only after confirming their informed consent. Because the sample was not selected through random sampling and was limited to a small number of institutions within a specific regional context, the representativeness of the sample may be limited.

The inclusion criteria were as follows: (1) full-time enrolled medical students; (2) aged 18 years or older; (3) voluntary participation in the study with signed informed consent; and (4) ability to independently read and comprehend the Chinese-language questionnaire. The exclusion criteria were as follows: (1) students enrolled in non-medical programs; (2) students on academic leave or prolonged absence from campus; (3) individuals who had experienced a major life stressor within the preceding 3 months that could significantly affect psychological assessment outcomes; and (4) invalid respondents whose questionnaire completion time fell below the minimum reasonable response time threshold. Participants were excluded if they reported experiencing a major life stressor within the previous 3 months. In the present study, this criterion was assessed through a self-reported screening item rather than a standardized validated life-event scale. A major life stressor was operationally defined as a significant adverse event that could substantially affect psychological status, including but not limited to bereavement, serious illness or injury, major family disruption, severe interpersonal conflict, serious academic or financial crisis, or other events perceived by the participant as highly stressful. The four psychological scales used in the present study comprised a total of 73 core items. Following the predefined criterion of 3 s per core item, the minimum reasonable completion time was calculated as 219 s. Questionnaires completed in less than 219 s were regarded as having insufficient response time and were excluded from the analysis.

Formal *a priori* power analysis for latent profile analysis (LPA) is not straightforward because sample size requirements depend on several design and model-related factors, including the number of profile indicators, class separation, class proportions, model complexity, and estimation stability. In the present study, the LPA was conducted using the 15 items of the Mindful Attention Awareness Scale as profile indicators. Therefore, the previously cited recommendation for LPA models with two to four indicators was not retained as a direct justification for the present analysis. Given the exploratory nature of the LPA and the use of item-level indicators, we adopted a pragmatic recruitment target of at least 400 participants and assessed the adequacy of the sample after model estimation using convergence status, information criteria, entropy, average posterior classification probabilities, and the size of the smallest latent profile. To allow for potentially invalid or incomplete questionnaires, the target sample size was increased by 5%, resulting in a planned recruitment target of 420 participants. The final valid sample included 460 participants, exceeding the planned target. However, we acknowledge that the use of 15 item-level indicators increases model complexity, and the sample size justification should therefore be interpreted as pragmatic.

During the data collection phase, a total of 494 participants were recruited. Among these, 16 participants declined to sign the informed consent form, 3 participants were under 18 years of age, 10 participants were enrolled in non-medical programs, and 5 completed the questionnaire in less than the predefined minimum reasonable completion time of 219 s. Consequently, the final valid sample comprised 460 participants, yielding an effective response rate of 93.12%. Of these, 241 were male (52.4%) and 219 were female (47.6%). The mean age of participants was 21.15 ± 1.304 years. Detailed demographic information is presented in Table 1.

**Table 1:** Demographic characteristics of participants.

Variables	Items	Number (N)	Proportion (%)
Gender	Male	241	52.4%
	Female	219	47.6%
Program duration	Four year program	31	6.7%
	Five year program	429	93.3%
Educational stage	Freshman/Sophomore year (Basic study)	58	12.6%
	Junior year/senior Year (Probation)	345	75.0%
	Fifth year (Internship stage)	57	12.4%
Only-child status	No	307	66.7%
	Yes	153	33.3%
Place of origin	Cities	328	71.3%
	Rural areas	132	28.7%
Weekly study hours	20 h and below	61	13.3%
	21–30 h	101	22.0%
	31–40 h	244	53.0%
	41–50 h	36	7.8%
	51 h and above	18	3.9%
Clinical practicum or clerkship experience	No	66	14.3%
	Yes	394	85.7%
Research and innovation experience	Participated but not completed	132	28.7%
	Participate and complete	149	32.4%
	No relevant experience but willing to participate	179	38.9%
Daily sleep duration	6 h and below	17	3.7%
	6.1–9 h	426	92.6%
	9.1 h and above	17	3.7%
Weekly exercise frequency	≤1 times	107	23.3%
	2–4 times	313	68.0%
	5 times and above	40	8.7%

### 2.2 Measurement tools

#### 2.2.1 Creative trait motivation scale

The Creative Trait Motivation Scale was developed by Taylor and Kaufman (2021) and comprises 20 items across three dimensions: intrinsic motivation, extrinsic motivation, and amotivation. The scale was translated into Chinese version by Xu et al. (2025), and its cultural adaptability and reliability were validated in a university student sample. In the present study, this scale was used to assess creative trait motivation among medical students. Responses were scored on a 6-point Likert scale ranging from 1 = strongly disagree to 6 = strongly agree. A sample item is “I experience a strong sense of pleasure when engaging in innovative activities.” Because the amotivation dimension is theoretically distinct from intrinsic and extrinsic motivation and represents a lack of creative motivation, the present study treated creative trait motivation as a multidimensional construct. In the main analyses, we used a composite creative trait motivation index to maintain consistency with the original mediation model. However, to avoid overinterpreting this composite index as a homogeneous construct, we also conducted supplementary subscale-level analyses for intrinsic motivation, extrinsic motivation, and amotivation. In the sensitivity analysis involving reduced amotivation, amotivation was reverse-scored so that higher scores represented lower creative amotivation.

In this study, the Cronbach’s alpha coefficient for this scale was 0.851, intrinsic motivation (Cronbach’s *α* = 0.963), extrinsic motivation (Cronbach’s α = 0.948), and amotivation (Cronbach’s α = 0.944), indicating good internal consistency. A confirmatory factor analysis was conducted to evaluate the measurement structure of the scale. The three-factor model showed acceptable fit: χ^2^/df = 3.895, GFI = 0.874, AGFI = 0.838, RMSEA = 0.079, CFI = 0.950, TLI = 0.942. To further justify the use of the composite index, a second-order factor model was tested, in which intrinsic motivation, extrinsic motivation, and reverse-scored amotivation loaded on a higher-order creative trait motivation factor. The model fit was acceptable fit: χ^2^/df = 3.963, GFI = 0.874, AGFI = 0.838, RMSEA = 0.079, CFI = 0.949, TLI = 0.941. These results provided psychometric support for using the composite score as an overall creative trait motivation index while retaining the theoretical distinction among the three subdimensions.

#### 2.2.2 Mindful attention awareness scale

The Mindful Attention Awareness Scale (MAAS) was developed by Brown and Ryan (2003) and comprises 15 items with a unidimensional measurement structure. The scale focuses on the individual’s level of attention to and awareness of present-moment experience in daily life. Item content pertains to attentional focus, automatized behavior, and awareness of physical and psychological states. A sample item is “I tend to rush through activities without being really attentive to them.” The scale was translated into Chinese by Chen et al. (2012), and its cultural adaptability and reliability were validated in a Chinese university student sample. In the present study, this scale was used to measure mindful attention awareness among medical students. Responses were scored on a 6-point Likert scale ranging from 1 (almost always) to 6 (almost never). Total scores range from 15 to 90, with higher scores reflecting higher levels of mindful attention awareness. In this study, the Cronbach’s alpha coefficient was 0.971, indicating excellent internal consistency. A confirmatory factor analysis of a single variable was conducted using AMOS 31.0 software, and the results showed that the model fit was excellent (χ2/df = 3.446, GFI = 0.923, AGFI = 0.886, RMSEA = 0.073, CFI = 0.972, TLI = 0.964).

Importantly, the MAAS primarily captures attentional awareness and reduced automaticity in daily activities. It does not directly assess other mindfulness-related constructs such as acceptance, non-judgment, non-reactivity, decentering, or compassion. Therefore, all interpretations in the present study are restricted to attentional awareness rather than multidimensional mindfulness as conceptualized in broader theoretical frameworks. Accordingly, where related constructs (e.g., openness, non-reactivity, or decentering) are mentioned in interpreting the present findings, they are presented solely as theoretical considerations drawn from broader mindfulness frameworks and were not directly measured in this study; the empirical interpretations are confined to present-centered attentional awareness and reduced automaticity as operationalized by the MAAS.

#### 2.2.3 Cognitive flexibility scale

The Chinese version of the Cognitive Flexibility Scale was developed by Wang, Yang (Wang et al., 2016) and comprises 20 items with a unidimensional measurement structure. In the present study, this scale was used to assess cognitive flexibility among medical students. A sample item is “I do not know how to make decisions in a difficult situation.” Responses were scored on a 6-point Likert scale ranging from 1 (strongly disagree) to 6 (strongly agree). Total scores range from 20 to 120, with higher scores indicating higher levels of cognitive flexibility. In this study, the Cronbach’s alpha coefficient was 0.892, indicating good internal consistency. A confirmatory factor analysis of a single variable was conducted using AMOS 31.0 software, and the results showed that the model fit was excellent (χ2/df = 1.754, GFI = 0.888, AGFI = 0.854, RMSEA = 0.041, CFI = 0.948, TLI = 0.942).

#### 2.2.4 Achievement goal orientation scale

The Chinese version of the Achievement Goal Orientation Questionnaire developed by Lau and Lee (2008) was used to assess students’ achievement goal orientation. The questionnaire comprises 18 items across three dimensions: mastery goals, performance-approach goals, and performance-avoidance goals. Mastery goals reflect students’ emphasis on learning, understanding, and competence development; performance-approach goals reflect their tendency to demonstrate competence and achieve favorable performance relative to others; and performance-avoidance goals reflect their tendency to avoid failure or negative judgments of competence. A sample item is “My goal in this class is to get better grades than most students.” Responses were scored on a 6-point Likert scale ranging from 1 (strongly disagree) to 6 (strongly agree). This scale does not contain any reverse-measurement items. In the present study, the total score was used as an index of the overall strength of achievement goal endorsement in academic contexts rather than as a measure of a purely adaptive or mastery-oriented goal orientation. This distinction is important because the three subdimensions are theoretically heterogeneous and performance-avoidance goals should not be interpreted as equivalent to mastery goals. To justify the use of the total score, we conducted confirmatory factor analyses to examine whether a higher-order measurement structure could adequately represent the three achievement goal components. Total scores range from 18 to 108, with higher scores indicating stronger achievement goal orientation. In this study, the Cronbach’s alpha coefficient was 0.882, mastery goals, performance-approach goals (Cronbach’s *α* = 0.941), and performance-avoidance goals (Cronbach’s *α* = 0.948). Indicating good internal consistency. A confirmatory factor analysis of a single variable was conducted using AMOS 31.0 software, and the results showed that the model fit was excellent (χ2/df = 1.741, GFI = 0.951, AGFI = 0.933, RMSEA = 0.040, CFI = 0.978, TLI = 0.975).

### 2.3 Data analysis

#### 2.3.1 Routine analysis

Data analyses in this study were performed using SPSS 27.0 and Mplus 8.3. The online questionnaire system required responses to all core scale items before submission; therefore, the final analytic dataset contained no item-level missing values for the core study variables. Prior to the main analyses, data were screened for response validity, range errors, distributional characteristics, and potential outliers.

Prior to the main analyses, data were screened for response validity, range errors, distributional characteristics, and potential outliers. First, all item responses were checked to confirm that they fell within the theoretically possible response range of the corresponding 6-point Likert-type scales. Second, questionnaire completion time was used as a response-quality screening criterion. Because the four psychological scales contained 73 core items in total, and the predefined minimum reasonable response time was 3 s per item, questionnaires completed in less than 219 s were considered invalid and excluded before analysis. Third, univariate outliers for composite scale scores were evaluated using standardized z scores, with ∣*z*∣ > 3.29 used as a reference criterion for potentially extreme values. Multivariate outliers among the core continuous variables were evaluated using Mahalanobis distance with reference to the chi-square distribution at *p* < 0.001. No additional cases were excluded solely on the basis of statistical outlier diagnostics, because no values were outside the allowable scale range and the detected score distributions were substantively plausible for self-report psychological scale data. Thus, the final valid analytic sample comprised 460 participants.

Descriptive statistics were computed to characterize the sample and the core study variables. Means and standard deviations were used for continuous variables, whereas frequencies and percentages were used for categorical variables. For the core psychological variables, composite scores were calculated as mean item scores, with higher scores indicating higher levels of the corresponding construct. Distributional normality was evaluated using skewness and kurtosis. Following Kline (2023) recommendation, absolute skewness values below 3 and absolute kurtosis values below 8 were considered acceptable for the planned parametric analyses.

Bivariate associations among mindful attention awareness, cognitive flexibility, creative trait motivation, and achievement goal orientation were examined using Pearson product–moment correlation coefficients because the analyses were based on composite scale scores rather than individual ordinal items. The direction, magnitude, and statistical significance of the correlations were reported. Correlation analyses were used primarily to provide preliminary descriptive evidence for theoretically specified associations among the study variables.

Between-group differences in achievement goal orientation across demographic characteristics were examined using independent-samples t-tests for dichotomous variables and one-way analyses of variance for variables with three or more categories. Effect sizes were reported as Cohen’s *d* for independent-samples t-tests and eta-squared (η^2^) for one-way analyses of variance. When an omnibus ANOVA was statistically significant, Bonferroni-adjusted pairwise comparisons were conducted to identify specific group differences while controlling for multiple comparisons. For dichotomous comparisons, two-tailed *p*-values were reported. Statistical significance was set at *p* < 0.05.

The cross-sectional sequential indirect association model was tested using PROCESS Model 6 with 5,000 bias-corrected bootstrap samples. Mindful attention awareness was entered as the independent variable, achievement goal orientation as the dependent variable, and cognitive flexibility and creative trait motivation as sequential mediators. Given the cross-sectional nature of the data, the model was interpreted as a theory-informed statistical indirect association model rather than as evidence of causal mediation. Gender, educational stage, only-child status, weekly study hours, and clinical practicum or clerkship experience were included as covariates because these variables showed significant between-group differences in achievement goal orientation in the preliminary analyses.

Categorical covariates were coded as follows. Gender was coded as male = 0 and female = 1. Only-child status was coded as no = 0 and yes = 1. Clinical practicum or clerkship experience was coded as no = 0 and yes = 1. Educational stage was dummy coded, with Junior/Senior Year, namely the probation stage, used as the reference category because it represented the largest subgroup. Two dummy variables were created: Freshman/Sophomore Year versus Junior/Senior Year, and Fifth year internship stage versus Junior/Senior Year. Weekly study hours were also dummy coded, with 31–40 h used as the reference category because it represented the largest subgroup. Four dummy variables were created to represent 20 h and below, 21–30 h, 41–50 h, and 51 h and above, respectively, relative to the 31–40 h reference group. Indirect associations were considered statistically significant when the 95% bootstrap confidence interval did not include zero.

For comparisons among latent profiles, most likely profile membership from the selected latent profile model was used as the grouping variable. One-way analyses of variance were conducted to compare cognitive flexibility, creative trait motivation, and achievement goal orientation across the identified profiles. Eta-squared (η^2^) was reported as the effect size. When the omnibus test was significant, Bonferroni-adjusted *post hoc* comparisons were used to examine pairwise differences among profiles, thereby reducing the risk of inflated Type I error due to multiple comparisons.

Because students were recruited from three medical schools and classes were used as sampling units, observations might not have been fully independent. Ideally, class-level intraclass correlation coefficients and cluster-robust standard errors should be used to evaluate and adjust for possible within-class clustering. However, because the questionnaire was administered anonymously and detailed class identifiers were not retained in the analytic dataset, we were unable to calculate class-level ICCs or conduct class-level cluster-robust sensitivity analyses. In addition, because only three schools were included, school-level multilevel modeling or school-level cluster-robust estimation was not statistically stable. This issue is acknowledged as a limitation of the present study.

#### 2.3.2 Latent profile analysis procedure

Latent profile analysis was conducted in Mplus 8.3 to examine whether medical students could be grouped into empirically distinguishable profiles based on their responses to the 15 MAAS items. Because the MAAS is a unidimensional scale, the profiles were expected to primarily reflect relative level differences in mindful attention awareness rather than multidimensional shape differences across different mindfulness facets. Therefore, the LPA results were interpreted as level-based profiles of mindful attention awareness.

All 15 MAAS items were scored in the same direction, with higher scores indicating higher mindful attention awareness. Because the items were measured on the same 6-point Likert response scale and represented the same construct, raw item scores were used as profile indicators rather than z-standardized scores. The final analytic dataset contained no missing responses on the MAAS items; therefore, no imputation was required for the LPA. Models were estimated using maximum likelihood estimation in Mplus. Following a conventional LPA specification, within-profile item variances were constrained to be equal across profiles, and within-profile covariances were fixed to zero; thus, local independence was assumed conditional on latent profile membership.

One- through five-profile models were estimated. To reduce the likelihood of local maxima, each model was estimated with 1,000 random sets of starting values and 200 final-stage optimizations. Model selection was based on a combination of statistical fit, classification quality, parsimony, and substantive interpretability. Specifically, Akaike information criterion, Bayesian information criterion, sample-size-adjusted Bayesian information criterion, entropy, the Lo–Mendell–Rubin adjusted likelihood ratio test, and the bootstrap likelihood ratio test were considered. Lower AIC, BIC, and aBIC values indicate better relative model fit, whereas entropy values closer to 1 indicate clearer classification. The LMR and BLRT were used to compare the k-profile model with the k − 1 profile model. In addition, the substantive meaning of the extracted profiles and the extent to which an additional profile represented a theoretically interpretable pattern rather than a further subdivision of overall severity or level were considered. Classification uncertainty was evaluated using entropy and average posterior classification probabilities for the selected solution.

## 3 Result

### 3.1 Common method bias

Given that this study employed a cross-sectional self-report questionnaire to collect variable data, despite researchers having adopted measures such as confidentiality assurance, anonymity, and reverse-scored items to mitigate common method bias, the potential risk of systematic interference with research findings could not be entirely eliminated. Accordingly, Harman’s single-factor test was conducted by subjecting all measurement items across the scales to an unrotated exploratory factor analysis. The first unrotated factor explained 23.385% of the total variance, which was below the commonly used 40% reference value. This finding suggests that no single factor accounted for the majority of covariance among the study variables. Nevertheless, Harman’s single-factor test has limited sensitivity and should not be interpreted as definitive evidence that common method bias is absent.

### 3.2 Descriptive statistics and correlation analysis

Mindful attention awareness, creative trait motivation, cognitive flexibility, and achievement goal orientation were all at moderately high levels. The skewness values of the core study variables ranged from −1.947 to −0.007, and the kurtosis values ranged from −0.486 to 6.356, none of which exceeded the thresholds proposed by Kline (2023) (skewness < |3|, kurtosis < |8|), indicating that the data distributions generally satisfied the assumption of normality.

As shown in Table 2, mindful attention awareness exhibited significant but weak positive correlations with cognitive flexibility (*r* = 0.322, *p* < 0.001), creative trait motivation (*r* = 0.226, p < 0.001), and achievement goal orientation (*r* = 0.143, *p* < 0.01). Creative trait motivation showed a significant weak positive correlation with cognitive flexibility (*r* = 0.209, *p* < 0.001) and a significant strong positive correlation with achievement goal orientation (*r* = 0.600, *p* < 0.001). Cognitive flexibility demonstrated a significant weak positive correlation with achievement goal orientation (*r* = 0.199, *p* < 0.001).

**Table 2:** Descriptive statistics and correlation analysis of core study variables.

Variables	*M*	SD	Skewness	Kurtosis	1	2	3	4
1. Mindful attention awareness	3.970	1.200	−0.604	−0.486	1			
2. Creative trait motivation	3.814	0.594	−0.380	1.621	0.226***	1		
3. Cognitive flexibility	3.275	0.562	−1.947	6.356	0.322***	0.209***	1	
4. Achievement goal orientation	3.658	0.660	−0.007	1.179	0.143**	0.600***	0.199***	1

Values for the psychological variables represent mean item scores. The possible range for each reported scale score was 1–6. **p < 0.01; ***p < 0.001.

### 3.3 Differences in achievement goal orientation across demographic characteristics

To systematically examine distributional differences in achievement goal orientation among medical students with varying demographic characteristics, independent-samples *t*-tests were employed for dichotomous variables and one-way analyses of variance (ANOVA) were conducted for polytomous variables. The specific results are presented in Table 3. The findings revealed that gender (*t* = −3.554, *p* < 0.001), educational stage (*F* = 10.447, *p* < 0.001), only-child status (*t* = −3.700, *p* < 0.001), weekly study hours (*F* = 5.327, *p* < 0.001), and clinical practicum or clerkship experience (*t* = 2.756, *p* = 0.006) were associated with significant differences in achievement goal orientation. In contrast, program duration (*t* = 1.047, *p* = 0.295), place of origin (*t* = −1.389, *p* = 0.166), research and innovation experience (*F* = 2.504, *p* = 0.083), daily sleep duration (*F* = 1.583, *p* = 0.206), and weekly exercise frequency (*F* = 2.413, *p* = 0.091) did not yield statistically significant between-group differences in achievement goal orientation.

**Table 3:** Differences in achievement goal orientation across demographic characteristics.

Variables	Items	*M*	SD	t/F	*p*	Cohen’s *d* / η^2^
Gender	Male	3.555	0.711	−3.554	< 0.001	−0.332
	Female	3.771	0.580			
Program duration	Four year program	3.778	0.698	1.047	0.295	0.195
	Five year program	3.649	0.657			
Educational stage	Freshman/Sophomore year (Basic study)	3.982	0.600	10.447	< 0.001	0.044
	Junior year/Senior year (Probation)	3.584	0.652			
	Fifth year (Internship stage)	3.774	0.665			
Only-child status	No	3.579	0.656	−3.700	< 0.001	−0.366
	Yes	3.817	0.641			
Place of origin	Cities	3.631	0.704	−1.389	0.166	−0.143
	Rural areas	3.725	0.532			
Weekly study hours	20 h and below	3.673	0.641	5.327	< 0.001	0.045
	21–30 h	3.727	0.573			
	31–40 h	3.554	0.675			
	41–50 h	3.944	0.619			
	51 h and above	4.056	0.745			
Clinical practicum or clerkship experience	No	3.864	0.507	2.756	0.006	0.367
	Yes	3.623	0.677			
Research and innovation experience	Participated but not completed	3.590	0.786	2.504	0.083	0.011
	Participate and complete	3.615	0.641			
	No relevant experience but willing to participate	3.743	0.562			
Daily sleep duration	6 h and below	3.892	0.692	1.583	0.206	0.007
	6.1–9 h	3.643	0.660			
	9.1 h and above	3.801	0.608			
Weekly exercise frequency	≤1 times	3.767	0.674	2.413	0.091	0.010
	2–4 times	3.612	0.651			
	5 times and above	3.721	0.669			

Independent-samples *t*-tests were used for dichotomous variables, and one-way ANOVA was used for variables with three or more categories. When the omnibus ANOVA was significant, Bonferroni-adjusted pairwise comparisons were conducted. Cohen’s d was reported for dichotomous comparisons and eta-squared (η^2^) for ANOVA models.

For multi-category demographic variables with significant omnibus ANOVA results, Bonferroni-adjusted pairwise comparisons were conducted to identify specific pairwise differences. For educational stage, students in the Freshman/Sophomore Year, Basic Study stage reported significantly higher achievement goal orientation than those in the Junior/Senior Year, Probation Stage. Other pairwise comparisons were not significant.

For weekly study hours, students studying 41–50 h per week reported significantly higher achievement goal orientation than those studying 31–40 h per week. Students studying 51 h or more per week also reported significantly higher achievement goal orientation than those studying 31–40 h per week. Other pairwise comparisons were not significant.

### 3.4 Cross-sectional sequential indirect association analysis involving cognitive flexibility and creative trait motivation

**Figure 1:**
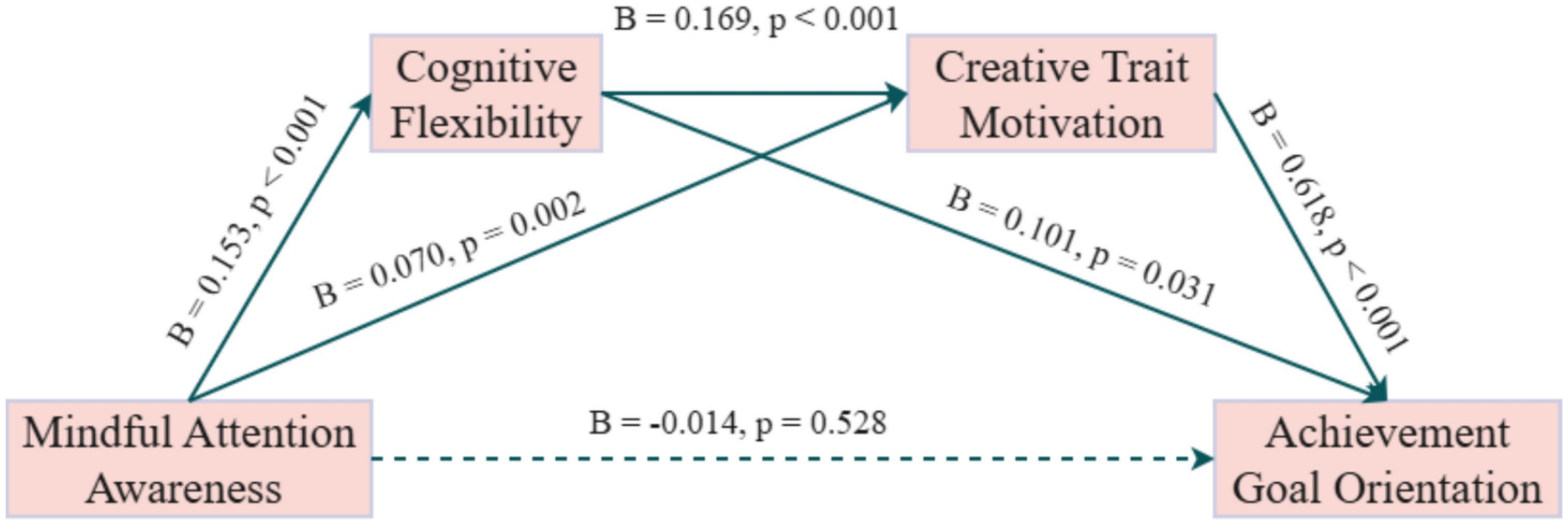
Path coefficients of the theory-informed cross-sectional sequential indirect association model.

The sequential mediation effect of cognitive flexibility and creative trait motivation was tested using the SPSS PROCESS macro (Model 6). In the model specification, mindful attention awareness was entered as the independent variable, achievement goal orientation as the dependent variable, and cognitive flexibility and creative trait motivation as mediators. Gender, educational stage, only-child status, weekly study hours, and clinical practicum experience were included as covariates. The statistical significance of the mediation effects was evaluated using the bias-corrected bootstrap method with 5,000 resamples. Because the data were cross-sectional, the model was interpreted as a statistical indirect association model rather than as evidence of causal mediation. The regression analysis results are presented in Table 4 and Figure 1.

**Table 4:** Regression analysis results for the cross-sectional sequential indirect association model involving cognitive flexibility and creative trait motivation.

Model	Outcome	Predictor	*R* ^2^	*F*	*B*	*t*	95% CI
Model 1	Cognitive flexibility	Mindful attention awareness	0.109	9.217	0.153	7.262***	[0.111, 0.194]
Model 2	Creative trait motivation	Mindful attention awareness	0.148	11.173	0.070	3.056**	[0.025, 0.115]
		Cognitive flexibility			0.169	3.471***	[0.073, 0.264]
Model 3	Achievement goal orientation	Mindful attention awareness	0.387	35.542	−0.014	−0.632	[−0.057, 0.029]
		Cognitive flexibility			0.101	2.163*	[0.009, 0.192]
		Creative trait motivation			0.619	13.924***	[0.531, 0.706]

***p < 0.001; **p < 0.01; *p < 0.05.

The results indicated that mindful attention awareness significantly and positively associated with cognitive flexibility (*B* = 0.153, *p* < 0.001, 95% CI = [0.111, 0.194]). Both mindful attention awareness (*B* = 0.070, *p* = 0.002, 95% CI = [0.025, 0.115]) and cognitive flexibility (*B* = 0.169, *p* < 0.001, 95% CI = [0.073, 0.264]) exerted significant positive associated with creative trait motivation. Cognitive flexibility (*B* = 0.101, *p* = 0.031, 95% CI = [0.009, 0.192]) and creative trait motivation (*B* = 0.619, *p* < 0.001, 95% CI = [0.531, 0.706]) both significantly and positively associated with achievement goal orientation. Notably, after simultaneously controlling for both mediators, the direct association of mindful attention awareness on achievement goal orientation was no longer statistically significant (*B* = −0.014, *p* = 0.528, 95% CI = [−0.057, 0.029]).

The decomposition of the sequential mediation effects of cognitive flexibility and creative trait motivation is presented in Table 5. The indirect association of Path 1 (mindful attention awareness → cognitive flexibility → achievement goal orientation) was 0.015 (SE = 0.008, 95% CI = [0.005, 0.033]), indicating that cognitive flexibility served as a significant mediator, thereby supporting Hypothesis H2. The indirect association of Path 2 (mindful attention awareness → creative trait motivation → achievement goal orientation) was 0.043 (SE = 0.016, 95% CI = [0.013, 0.077]), indicating that creative trait motivation served as a significant independent mediator, thereby supporting Hypothesis H3. The indirect association of Path 3 (mindful attention awareness → cognitive flexibility → creative trait motivation → achievement goal orientation) was 0.016 (SE = 0.006, 95% CI = [0.005, 0.029]), indicating that cognitive flexibility and creative trait motivation together constituted a significant theory-informed sequential indirect pathway, thereby supporting Hypothesis H4.

**Table 5:** Decomposition of statistical indirect associations in the cross-sectional theory-informed sequential indirect association model.

Types of effect	Effect	SE	LLCI	ULCI
Total effect	0.061	0.025	0.012	0.109
Direct effect	−0.014	0.022	−0.057	0.029
Indirect effect	0.075	0.019	0.038	0.113
Ind 1	0.015	0.008	0.005	0.033
Ind 2	0.043	0.016	0.013	0.077
Ind 3	0.016	0.006	0.005	0.029

Ind 1: Mindful attention awareness → Cognitive flexibility → Achievement goal orientation; Ind 2: Mindful attention awareness → Creative trait motivation → Achievement goal orientation; Ind 3: Mindful attention awareness → Cognitive flexibility → Creative trait motivation → Achievement goal orientation.

More importantly, the indirect association of mindful attention awareness on achievement goal orientation was negative and did not reach statistical significance, revealing an inconsistency in directionality between the direct and indirect effects. This pattern suggests the possible presence of a suppression effect within the model. After controlling for the cognitive and motivational pathways, the originally weak total positive correlation between mindful attention awareness and achievement goal orientation (*r* = 0.143, *p* < 0.001) exhibited a negative directional trend.

### 3.5 Sensitivity analyses using creative trait motivation subscales

To address the multidimensional nature of the Creative Trait Motivation Scale, supplementary subscale-level analyses were conducted for intrinsic motivation, extrinsic motivation, and amotivation. As shown in Table 6, intrinsic motivation was significantly and positively correlated with mindful attention awareness (*r* = 0.147, *p* < 0.01), cognitive flexibility (*r* = 0.179, *p* < 0.01), and achievement goal orientation (*r* = 0.294, *p* < 0.01). Extrinsic motivation was significantly correlated with mindful attention awareness (*r* = 0.193, *p* < 0.01) and achievement goal orientation (*r* = 0.478, *p* < 0.01), but its correlation with cognitive flexibility was not significant (*r* = 0.090). Amotivation was not significantly correlated with mindful attention awareness (*r* = 0.001) or cognitive flexibility (*r* = 0.057), but was positively correlated with achievement goal orientation (*r* = 0.250, *p* < 0.01).

**Table 6:** Descriptive statistics and correlations among creative trait motivation subscales and core study variables.

Variables	*M*	SD	1	2	3	4	5	6
Mindful attention awareness	3.970	1.200	1					
Cognitive flexibility	3.275	0.562	0.322**	1				
Achievement goal orientation	3.658	0.660	0.143**	0.199**	1			
Intrinsic motivation	4.453	0.892	0.147**	0.179**	0.294**	1		
Extrinsic motivation	3.353	1.181	0.193**	0.09	0.478**	−0.140**	1	
Amotivation	2.909	1.110	0.001	0.057	0.250**	−0.333**	0.565**	1

**p < 0.01.

We further repeated the sequential indirect association analyses by replacing the composite creative trait motivation index with each subscale. As shown in Table 7, the sequential indirect pathway through intrinsic motivation was significant: mindful attention awareness → cognitive flexibility → intrinsic motivation → achievement goal orientation, effect = 0.007, SE = 0.003, 95% CI [0.002, 0.013]. In contrast, the corresponding pathway through extrinsic motivation was not significant, effect = 0.003, SE = 0.004, 95% CI [−0.005, 0.012]. The pathway through reverse-scored amotivation was also not significant, effect = 0.003, SE = 0.003, 95% CI [−0.002, 0.009].

**Table 7:** Subscale-level sensitivity analyses of sequential indirect associations.

Model	Indirect pathway	Effect	SE	95% CI
Intrinsic motivation model	MAA → CF → intrinsic motivation → AGO	0.007	0.003	[0.002,0.013]
Extrinsic motivation model	MAA → CF → extrinsic motivation → AGO	0.003	0.004	[−0.005,0.012]
Amotivation model	MAA → CF → reversed amotivation → AGO	0.003	0.003	[−0.002,0.009]

MAA = mindful attention awareness; CF = cognitive flexibility; AGO = achievement goal orientation. Confidence intervals were estimated using 5,000 bootstrap samples. An indirect association was considered statistically significant when the 95% confidence interval did not include zero.

### 3.6 Latent profile analysis of mindful attention awareness

To further explore the within-group heterogeneity structure of mindful attention awareness among medical students, this study used the 15 measurement items of the Mindful Attention Awareness Scale as continuous profile indicator variables and fitted one- through five-class latent profile models in Mplus 8.3 using maximum likelihood estimation. To prevent maximum likelihood estimation from converging on local optima, each candidate model was specified with 1,000 sets of random starting values (starts = 1,000 200), and the solution yielding the largest log-likelihood value was selected as the final estimate. The fit indices for each candidate model are presented in Table 8.

**Table 8:** Fit indices for the one- through five-class latent profile models of mindful attention awareness.

Profile	AIC	BIC	aBIC	Entropy	LMR (p)	BLRT (p)	Proportions
1	24463.430	24587.367	24492.155	–	–	–	–
2	20293.979	20484.016	20338.025	0.963	0.005	<0.001	35.22%/64.78%
**3**	**18540.092**	**18796.228**	**18599.457**	**0.961**	**<0.001**	**<0.001**	**17.64%/43.38%/38.98%**
4	17891.402	18213.638	17966.088	0.951	<0.001	<0.001	13.69%/23.04%/33.04%/30.22%
5	17663.817	18052.152	17753.823	0.929	0.057	<0.001	11.30%/12.17%/24.13%/24.78%/27.61%

AIC = Akaike information criterion; BIC = Bayesian information criterion; aBIC = sample-size-adjusted Bayesian information criterion; LMR = Lo–Mendell–Rubin adjusted likelihood ratio test; BLRT = bootstrap likelihood ratio test. The 15 MAAS items were used as continuous indicators. The three-profile solution was selected based on fit indices, entropy, parsimony, and substantive interpretability. Bold values indicate the optimal three-profile solution selected for further analysis based on fit indices, entropy, parsimony, and substantive interpretability.

Model comparison results indicated that as the number of profiles increased, all three information criteria exhibited a continuous declining trend, reflecting progressively improved model fit. From the one-profile to the two-profile model, information criteria decreased substantially (ΔAIC = 4,169.451, ΔBIC = 4,103.351), both the LMR test (*p* = 0.005) and the BLRT (*p* < 0.001) reached statistical significance, and the Entropy value was as high as 0.963, indicating that the two-profile model demonstrated a significant fit advantage over the one-profile model. From the two-profile to the three-profile model, information criteria continued to improve substantially (ΔAIC = 1,753.887, ΔBIC = 1,687.788), both the LMR test (*p* < 0.001) and the BLRT (*p* < 0.001) were significant, and the Entropy value of 0.961 remained at an excellent level. From the three-profile to the four-profile model, the magnitude of improvement in information criteria notably decelerated (ΔAIC = 648.690, ΔBIC = 582.590); although the LMR (*p* < 0.001) and BLRT (*p* < 0.001) remained significant, the marginal benefit of model improvement had diminished considerably. From the four-profile to the five-profile model, the decline in information criteria further narrowed (ΔAIC = 227.585, ΔBIC = 161.486), and the LMR test did not reach statistical significance (*p* = 0.057), indicating that the five-profile model failed to provide a significant improvement in fit over the four-profile model.

Although the four-profile model also showed lower information criteria and significant likelihood ratio tests, several considerations favored the three-profile solution. First, the three-profile solution already demonstrated excellent classification quality, with an entropy value of 0.961. Second, the improvement in information criteria from the three-profile to the four-profile model was much smaller than the improvement from the two-profile to the three-profile model, suggesting diminishing marginal gains in model fit. Third, inspection of the profile pattern indicated that the four-profile solution mainly subdivided the same overall level continuum of mindful attention awareness rather than yielding a clearly distinct and theoretically meaningful profile pattern. Fourth, the three-profile solution was more parsimonious and more directly interpretable as low, moderate, and high mindful attention awareness profiles. Therefore, based on statistical adequacy, classification quality, parsimony, and substantive interpretability, the three-profile solution was retained, thereby supporting Hypothesis H5.

The average posterior classification probabilities are presented in Table 9. The diagonal average posterior probability of the three categories was higher than 0.98, and the off-diagonal probability was very low (≤ 0.016), indicating that the model could classify individuals into their categories with high accuracy.

**Table 9:** Average posterior classification probabilities for the selected three-profile solution.

Most likely latent profile	Low	High	Medium
Low	0.985	0.000	0.015
High	0.000	0.984	0.016
Medium	0.002	0.016	0.982

Low = Low mindful attention awareness group; High = High mindful attention awareness group; Medium = Medium mindful attention awareness group.

**Figure 2:**
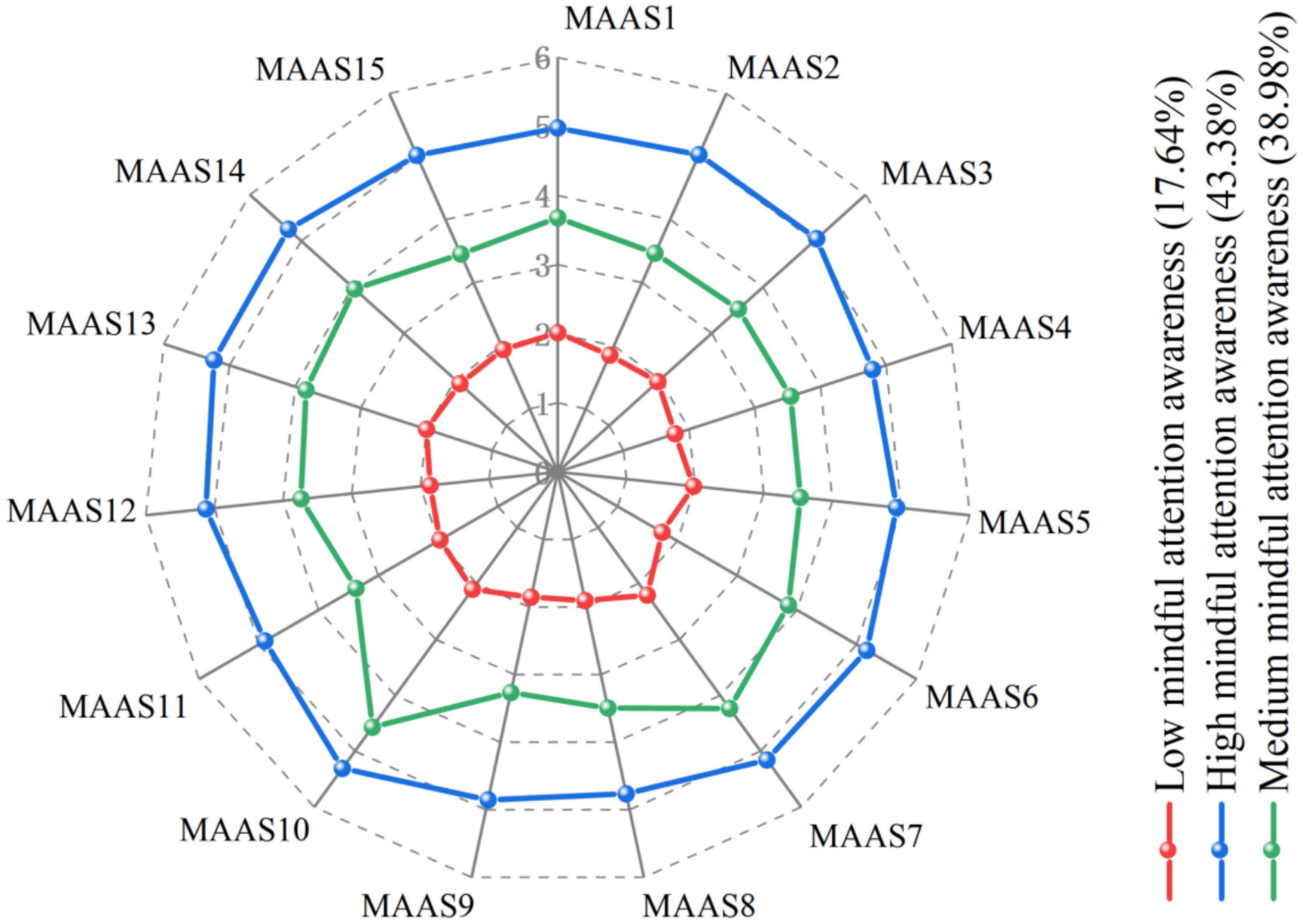
Profile plot of the three-class latent profile model.

Based on the analytical results in Table 6, the profile plot for the three-class latent profile model was generated using Origin 2021 software, as shown in Figure 2. Profile 1 was labeled the “low mindful attention awareness group” (*n* = 81, 17.64%), indicating that these individuals exhibited relatively weak attention and awareness of present-moment experience in daily study and life, and were more susceptible to automatic response patterns and attentional dispersion. Profile 2 was labeled the “high mindful attention awareness group” (*n* = 200, 43.38%), suggesting that these individuals possessed relatively strong present-centered attention and awareness and were able to maintain conscious attention to present-moment experience with reduced automaticity in daily contexts. Profile 3 was labeled the “moderate mindful attention awareness group” (*n* = 179, 38.98%), exhibiting moderate levels of mindful attention awareness characteristics.

### 3.7 Differences among optimal profiles in cognitive flexibility, creative trait motivation, and achievement goal orientation

**Figure 3:**
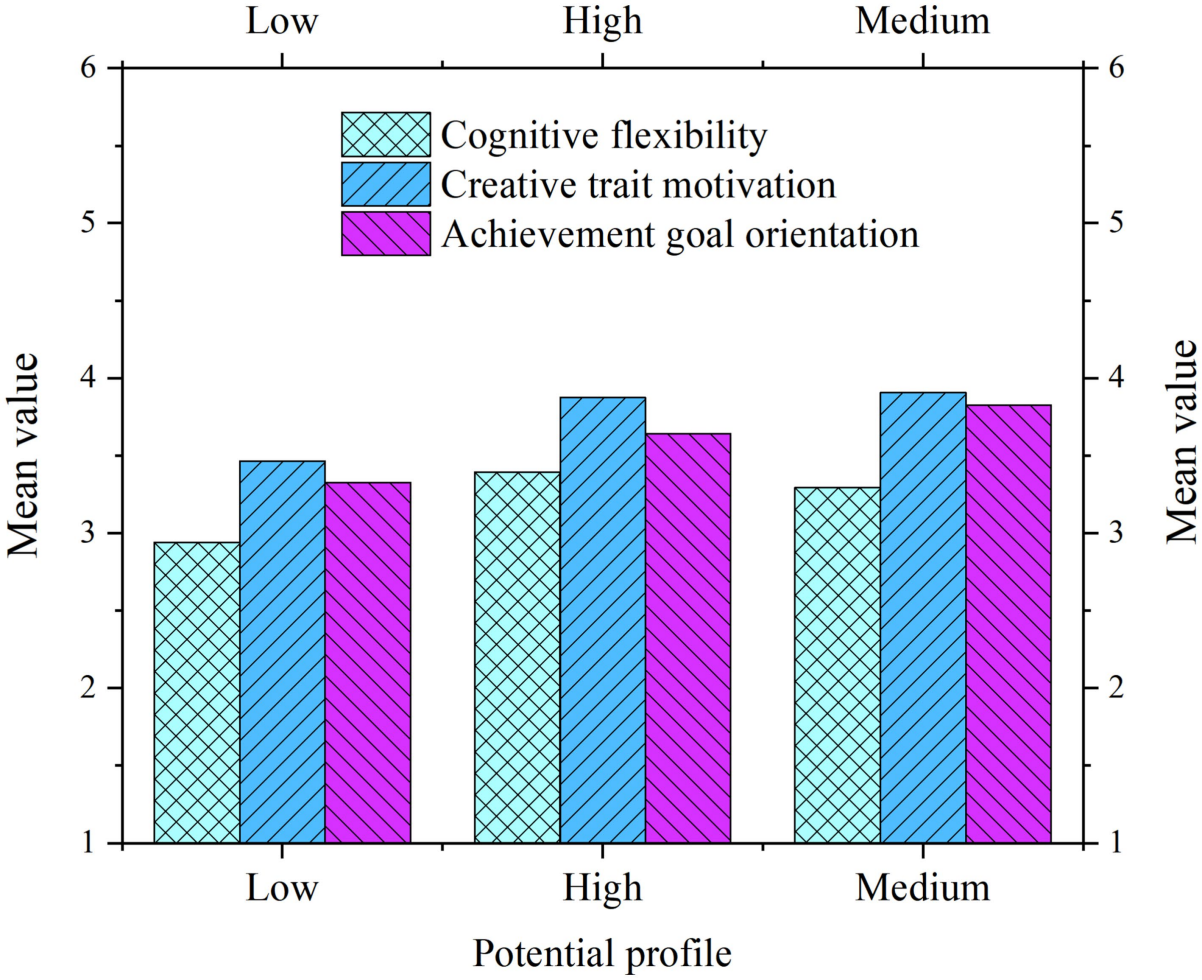
Differences among optimal profiles in cognitive flexibility, creative trait motivation, and achievement goal orientation.

Using the latent profile classification results as the grouping variable, one-way analyses of variance were conducted with cognitive flexibility, creative trait motivation, and achievement goal orientation as the respective dependent variables. The results are presented in Table 10 and Figure 3. The optimal three profiles of mindful attention awareness demonstrated significant and substantively meaningful differences in cognitive flexibility (*F* = 20.589, *p* < 0.001, η^2^ = 0.083), creative trait motivation (*F* = 18.599, *p* < 0.001, η^2^ = 0.075), and achievement goal orientation (*F* = 17.337, *p* < 0.001, η^2^ = 0.071), thereby supporting Hypothesis H6.

**Table 10:** Differences among optimal profiles in cognitive flexibility, creative trait motivation, and achievement goal orientation.

Optimal profiles	Cognitive flexibility		Creative trait motivation		Achievement goal orientation	
	*M*	SD	*M*	SD	*M*	SD
Low	2.942 ^a^	0.970	3.466 ^a^	0.630	3.327 ^a^	0.773
High	3.394 ^b^	0.392	3.875 ^b^	0.599	3.643 ^b^	0.584
Medium	3.295 ^b^	0.383	3.906 ^b^	0.510	3.827 ^c^	0.627
*p*	<0.001		<0.001		<0.001	
*F*	20.589		18.599		17.337	
*η* ^2^	0.083		0.075		0.071	

Low = Low mindful attention awareness group; High = High mindful attention awareness group; Medium = Medium mindful attention awareness group. Different superscript letters within the same row indicate significant differences between groups based on Bonferroni-adjusted post hoc comparisons, Groups sharing the same superscript letter do not differ significantly.

The low mindful attention awareness profile showed the lowest scores on all three outcome variables. However, the pattern was not strictly monotonic across all outcomes. The high mindful attention awareness profile showed the highest level of cognitive flexibility, whereas the moderate mindful attention awareness profile showed slightly higher levels of creative trait motivation and achievement goal orientation than the high mindful attention awareness profile. This finding provides incremental evidence for the protective function of mindful attention awareness from a person-centered perspective. It simultaneously highlights the urgency and necessity of implementing targeted mindfulness interventions for medical students in the low mindful attention awareness group, facilitating their transition from low mindfulness states toward moderate or higher levels, thereby promoting comprehensive enhancement in cognitive flexibility, creative motivation, and adaptive achievement goal orientation.

Bonferroni-adjusted *post hoc* comparisons further clarified the pairwise differences among the three profiles. For cognitive flexibility, the low mindful attention awareness profile scored significantly lower than both the high and moderate profiles, whereas the high and moderate profiles did not differ significantly. The same pattern was observed for creative trait motivation: the low profile scored significantly lower than both the high and moderate profiles, while the high and moderate profiles did not differ significantly. For achievement goal orientation, the low mindful attention awareness profile scored significantly lower than both the high and moderate profiles. What is more, the high mindful attention awareness profile scored significantly lower than both the moderate profiles.

## 4 Discussion

### 4.1 Variable-centered analysis

The variable-centered findings showed that, after cognitive flexibility and creative trait motivation were simultaneously included in the model, the direct association between mindful attention awareness and achievement goal orientation was no longer statistically significant, whereas several theory-informed indirect associations were significant. This finding extends the existing theoretical understanding of the relationship between mindfulness and academic motivation in the literature, demonstrating that mindful attention awareness does not directly drive the formation of adaptive goal orientations but instead operates through two sequentially linked intermediate pathways involving cognitive processing and motivational activation.

Cognitive flexibility showed a significant statistical indirect effect in the association between mindful attention awareness and achievement goal orientation. According to attentional monitoring theory (Eysenck and Derakshan, 2011), mindful awareness is related to metacognitive monitoring of attentional allocation and reduced automatic processing (Kudesia, 2019). Within the context of medical education, the academic tasks confronting medical students are often characterized by a high degree of complexity and uncertainty (Weurlander et al., 2019). In this context, higher mindful attention awareness may be associated with reduced automatic reactivity and greater awareness of ongoing cognitive processes, which are also conceptually related to cognitive flexibility. In turn, students with higher cognitive flexibility may be more likely to reinterpret academic difficulties and evaluative challenges as opportunities for learning and competence development. Thus, the present findings provide preliminary cross-sectional evidence that cognitive flexibility may constitute a theory-relevant cognitive correlate linking mindful attention awareness with achievement goal orientation.

The Creative Trait Motivation Scale includes intrinsic motivation, extrinsic motivation, and amotivation, which should not be treated as theoretically identical components. Consistent with this multidimensional view, the present sensitivity analyses showed that the sequential indirect association was primarily supported by intrinsic creative motivation. Specifically, the pathway from mindful attention awareness to achievement goal orientation through cognitive flexibility and intrinsic motivation was significant, whereas the corresponding pathways through extrinsic motivation and reverse-scored amotivation were not significant. This pattern is theoretically meaningful. Intrinsic creative motivation reflects interest, enjoyment, curiosity, and self-endorsed engagement in creative activities. Medical students with higher mindful attention awareness may be more capable of noticing present-moment cognitive experiences and disengaging from automatic responses. This attentional awareness may support cognitive flexibility, which in turn may facilitate the perception of novelty, multiple possibilities, and self-directed exploration in learning tasks. These cognitive conditions are especially relevant for intrinsic creative motivation. Once intrinsic creative motivation is activated, students may be more likely to construe academic tasks as opportunities for exploration, growth, and competence development, thereby showing stronger achievement goal orientation. By contrast, extrinsic creative motivation was strongly correlated with achievement goal orientation but was not significantly associated with cognitive flexibility, and the sequential indirect pathway involving extrinsic motivation was not significant. This suggests that extrinsic creative motivation may be more closely related to evaluative concerns, external recognition, or performance-related striving than to the cognitive flexibility pathway proposed in the present model. Similarly, the pathway involving reverse-scored amotivation was not significant, indicating that the present sequential mechanism was not mainly explained by reduced creative amotivation.

These results indicate that the significant pathway involving the composite creative trait motivation index in the main model should be interpreted cautiously. Rather than suggesting that all components of creative trait motivation operate equivalently, the findings suggest that intrinsic creative motivation is the most theoretically relevant motivational component in the link among mindful attention awareness, cognitive flexibility, and achievement goal orientation. This revision improves the conceptual precision of the model and aligns the interpretation more closely with the multidimensional structure of creative trait motivation.

### 4.2 Person-centered analysis

The findings revealed three qualitatively distinct subgroup types of mindful attention awareness among the medical student population. This confirms the within-group heterogeneity of mindful attention awareness among medical students from a person-centered perspective, indicating that the sample homogeneity assumption upon which traditional variable-centered analyses rely does not fully hold within the medical student population. The present study is the first to reveal the subgroup distributional characteristics of mindful attention awareness within the specific population of medical students, thereby extending the applicability and population coverage of latent profile research on mindful attention awareness.

In terms of profile distribution proportions, the high mindful attention awareness group accounted for the largest share (43.38%), followed by the moderate mindful attention awareness group (38.98%), with the low mindful attention awareness group comprising the smallest proportion (17.64%). This distributional pattern presents an overall positive outlook, indicating that the majority of medical students possess moderate or above levels of mindful attention awareness. Nevertheless, a subset of medical students remains in a state of low mindful attention awareness, suggesting that this subgroup may face greater risks of attentional dysregulation and automatic reactivity, warranting heightened attention from educators. The low mindful attention awareness group exhibited relatively weak attention and awareness capabilities toward present-moment experience in daily study and life, and was more prone to falling into habitual thinking patterns and emotional reactions. This psychological state may further exacerbate academic adaptation difficulties within the high-pressure environment of medical education.

It is noteworthy that participants in the medium mindfulness group reported higher achievement goal orientation and creative trait motivation than those in the high mindfulness group. One possible explanation is that the association between mindfulness and motivational factors may follow a nonlinear pattern. Moderate mindfulness may be associated with an optimal balance between awareness and goal-directed engagement. Individuals with moderate mindfulness may be sufficiently attentive and self-aware to regulate their cognition and emotion while still maintaining strong achievement striving, exploratory motivation, and creative engagement. By contrast, it is possible that higher levels of attentional awareness may be associated with a more process-focused learning style, which could be associated with less emphasis on achievement-oriented or performance-focused goals. Thus, the lower scores of the high mindfulness group on achievement goal orientation and creative trait motivation do not necessarily indicate a disadvantage, but may reflect a more process-oriented and less outcome-dependent motivational style. Nevertheless, this result should be interpreted cautiously. The mindfulness groups were created based on relative scores within the present sample, and the cut-off points were not externally validated. Moreover, because this study used a cross-sectional design, it remains unclear whether mindfulness influences motivational orientation or whether individuals with different motivational profiles differ in mindfulness levels. Future research should investigate nonlinear associations and examine the roles of specific mindfulness facets in achievement motivation and creative motivation.

More importantly, the different mindful attention awareness profiles exhibited significant differences in cognitive flexibility, creative trait motivation, and achievement goal orientation, providing incremental evidence for the protective function of mindful attention awareness from a person-centered perspective. Specifically, the low mindful attention awareness group scored significantly lower than both the high and moderate mindful attention awareness groups on all three outcome variables. The disadvantage of the low mindful attention awareness group in cognitive flexibility implies that this subgroup experiences greater difficulty in effective strategy adjustment and perspective shifting when confronting changing academic environments. Their weakness in creative trait motivation indicates insufficient intrinsic drive toward exploratory and innovative academic activities, while their lower scores in achievement goal orientation suggest that this subgroup may be more inclined to adopt avoidant or surface-level learning orientations. The synergistic attenuation of these three psychological functions constitutes an unfavorable psychological profile combination that may give rise to cumulative developmental disadvantages.

The present study integrated the results from both variable-centered and person-centered analytical strategies. The sequential mediation model revealed the process mechanism through which mindful attention awareness influences achievement goal orientation via cognitive flexibility and creative trait motivation, while the latent profile analysis further indicated that the operation of this process mechanism may manifest differentially depending on the category of mindful attention awareness level to which an individual belongs. The dual weakness of the low mindful attention awareness group in cognitive flexibility and creative trait motivation corresponds precisely to functional insufficiency at two critical transmission nodes in the sequential mediation model, which may serve as an important explanatory mechanism for the lower achievement goal orientation observed in this subgroup.

### 4.3 Practical implications and educational considerations

The present findings may provide several cautious educational implications for medical student development and support. First, mindful attention awareness may be considered a relevant psychological characteristic in the academic development and mental health promotion of medical students. Traditional medical education has tended to place greater emphasis on knowledge acquisition and skills training, whereas students’ attentional regulation, emotional awareness, and self-monitoring capacities in high-pressure learning environments may receive comparatively less attention. Based on the cross-sectional findings of the present study, mindful attention awareness was associated not only with cognitive flexibility and creative trait motivation but also with achievement goal orientation. These association patterns suggest that mindfulness-informed educational activities may be relevant to students’ academic and psychological development. However, because the present study adopted a cross-sectional design, these findings should not be interpreted as evidence that mindfulness training would directly change cognitive flexibility, creative trait motivation, or achievement goal orientation. Rather, they indicate that mindful attention awareness may be a meaningful focus for future longitudinal and intervention research in medical education.

Second, the observed theory-informed indirect associations suggest that educational support programs may benefit from considering cognitive, motivational, and goal-related factors together. In the present study, cognitive flexibility and creative trait motivation were statistically involved in the association between mindful attention awareness and achievement goal orientation. This pattern suggests that mindfulness-informed educational support could be considered alongside activities related to flexible thinking, multi-perspective problem solving, open-ended task exploration, and creative engagement. For instance, problem-based learning, case-based learning, clinical reasoning exercises, research training, and reflective learning activities may provide appropriate contexts in which students can practice perspective shifting, problem redefinition, and exploratory engagement. These suggestions should be understood as educational considerations derived from cross-sectional association patterns rather than as evidence-based intervention prescriptions. Future longitudinal or experimental studies are needed to determine whether integrating mindfulness-informed activities with cognitive flexibility training and creative learning experiences can contribute to changes in students’ motivational and academic goal-related outcomes.

Finally, the latent profile findings may help educators recognize heterogeneity in mindful attention awareness among medical students. The low mindful attention awareness profile was associated with lower levels of cognitive flexibility, creative trait motivation, and achievement goal orientation, suggesting that students with this profile may warrant additional attention in student support services. For these students, structured mindfulness-informed activities, focused attention exercises, stress identification and emotion regulation guidance, mentor feedback, and peer support could be considered as potentially useful forms of educational support. For students in the moderate mindful attention awareness profile, educational activities that encourage flexible thinking and creative engagement may be particularly relevant. For students in the high mindful attention awareness profile, support may focus on helping them apply their attentional awareness in academic, research, and clinical learning contexts. Nevertheless, these profile-based suggestions should be interpreted cautiously because latent profile membership was identified from cross-sectional data and does not establish developmental trajectories or intervention responsiveness. Future studies should examine whether profile-based educational support can be prospectively associated with improvements in cognitive, motivational, and academic outcomes.

These practical implications should be interpreted as preliminary and hypothesis-generating. The present findings indicate that mindful attention awareness, cognitive flexibility, creative trait motivation, and achievement goal orientation are interrelated among medical students, but they do not establish causal or temporal pathways. Therefore, the proposed educational considerations may inform future intervention design and longitudinal research, rather than providing definitive evidence for specific educational intervention effects.

### 4.4 Theoretical implications for medical education

This study contributes to the theoretical understanding of mindfulness, motivation, and creativity in the context of medical education. First, the findings extend mindfulness theory by suggesting that mindfulness may be associated not only with stress reduction and emotional regulation but also with creativity-related characteristics among medical students. Previous applications of mindfulness in medical education have often emphasized well-being, burnout prevention, and psychological adjustment (Scheepers et al., 2020). The present findings broaden this perspective by indicating that mindfulness may also be relevant to students’ creative development and motivational functioning.

Second, this study refines creativity theory by situating creativity within the highly structured and professionally demanding context of medical education. Unlike some general educational contexts, medical education requires students to develop creative thinking while also adhering to evidence-based reasoning, ethical standards, and clinical responsibility (Carrese et al., 2015). Therefore, creativity in medical students should not be understood simply as divergent thinking or novelty generation, but as adaptive, responsible, and context-sensitive problem solving. The observed associations among mindfulness, creative trait motivation, and achievement goal orientation suggest that creative development in medical education may depend on both self-regulatory awareness and motivational engagement.

Third, the findings also extend achievement goal theory by showing that achievement-oriented striving among medical students may be linked with mindfulness and creativity-related motivation. In particular, the finding that the medium mindfulness group showed higher achievement goal orientation and creative trait motivation than the high mindfulness group suggests that these relationships may not be strictly linear. Moderate mindfulness may represent a balance between self-awareness and active goal pursuit, whereas higher mindfulness may reflect a more process-oriented and less outcome-dependent motivational style. Thus, this study provides a more nuanced theoretical account of how mindfulness, motivation, and creativity may interact in medical education.

### 4.5 Limitations and future research directions

Despite the aforementioned findings, this study is not without limitations and shortcomings. The present study employed a cross-sectional survey design. Cross-sectional data can inherently provide only evidence of associations among variables and cannot rule out alternative explanations such as reverse causation and bidirectional influences. Future research should adopt longitudinal tracking designs or cross-lagged panel analyses, measuring core variables at multiple time points to more rigorously examine the temporal relationships and causal directions among variables. Additionally, experimental or quasi-experimental research designs would provide more compelling evidence for establishing causal mechanisms.

Although the final sample size exceeded the planned recruitment target, the LPA used 15 item-level indicators, which increased model complexity. Because formal power analysis for LPA is not straightforward and depends on factors such as class separation, profile proportions, and indicator distributions, the sample size justification was pragmatic rather than based on a definitive power calculation. Future studies with larger and more diverse samples should replicate the latent profile structure and examine its stability across independent samples.

Second, data collection in the present study relied entirely on self-report questionnaires, a measurement approach potentially susceptible to social desirability effects, self-perception biases, and recall biases. Although Harman’s single-factor test showed that the first unrotated factor explained 23.385% of the total variance, this test is only a preliminary diagnostic tool and has limited sensitivity in detecting method effects. Therefore, the possibility of common method bias cannot be completely ruled out. Future research could introduce multi-source evaluation data (such as teacher ratings and peer ratings), behavioral observation indicators, and physiological measures (such as attention-related neuroelectrophysiological indicators) as objective measurement tools to enhance measurement reliability and validity.

Another limitation concerns the assessment of recent major life stressors. In this study, the exclusion criterion of experiencing a major life stressor within the past 3 months was based on a self-reported screening item rather than a standardized validated assessment tool. Therefore, participants’ interpretation of this criterion may have varied, potentially leading to subjective bias or misclassification. Future research should use validated life-event inventories or structured interviews to ensure more reliable and standardized assessment of recent major stressors.

What is more, the limitation concerns the measurement scope of mindful attention awareness. The present study used the MAAS, which primarily assesses attention to and awareness of present-moment experiences and reduced automaticity. It does not directly measure other mindfulness-related facets, such as acceptance, non-judgment, non-reactivity, decentering, or compassion. Therefore, the findings should not be generalized to multidimensional mindfulness as a whole. Future studies should incorporate multidimensional mindfulness instruments, such as measures that separately assess observing, describing, acting with awareness, non-judging, and non-reactivity, to clarify whether different mindfulness facets have distinct relationships with cognitive flexibility, creative trait motivation, and achievement goal orientation.

Limitation concerns the clustered sampling structure. Although the schools were selected by convenience and classes were randomly selected within participating schools, the students were nested within schools and classes, which may have introduced within-cluster similarity and weakened the assumption of independent observations. Because detailed class identifiers were not retained in the analytic dataset, class-level ICCs and cluster-robust sensitivity analyses could not be conducted. In addition, because only three schools were included, school-level multilevel modeling was not statistically stable. Therefore, the possible influence of school- or class-level clustering cannot be fully ruled out. Future studies should recruit participants from a larger number of schools and classes, retain complete cluster identifiers, and use multilevel models or cluster-robust standard errors to account for the nested sampling structure.

A further limitation concerns the use of a composite creative trait motivation index. The Creative Trait Motivation Scale includes intrinsic motivation, extrinsic motivation, and amotivation, which are theoretically distinct components. Although the composite index was retained in the main model for consistency with the original analytic framework, supplementary subscale-level analyses showed that the sequential indirect association was primarily driven by intrinsic creative motivation, whereas the pathways involving extrinsic motivation and reverse-scored amotivation were not significant. Therefore, the composite score should not be interpreted as a homogeneous indicator of adaptive creative motivation. Future studies should further examine the distinct roles of intrinsic creative motivation, extrinsic creative motivation, and creative amotivation in medical education, especially using longitudinal or experimental designs.

Finally, the use of convenience sampling from only three regional medical schools limits the external validity of the findings. The participating students may not be representative of medical students from other geographic regions, institutional types, or educational contexts. In addition, convenience sampling may introduce selection bias, because students who agreed to participate may differ from those who did not. Therefore, the present results should be generalized with caution. Future studies should recruit larger and more diverse samples from multiple regions and institutions, preferably using random, stratified, or cluster sampling methods, to improve sample representativeness and further validate the generalizability of the findings.

## 5 Conclusion

The present study investigated 460 medical students using an integrated analytical approach that combined variable-centered and person-centered perspectives. The findings showed that mindful attention awareness was positively associated with cognitive flexibility, creative trait motivation, and achievement goal orientation. In the cross-sectional, theory-informed sequential indirect association model, cognitive flexibility and the composite creative trait motivation index were involved in significant statistical indirect pathways linking mindful attention awareness with achievement goal orientation. However, supplementary subscale-level analyses further showed that the sequential pathway through creative trait motivation was primarily attributable to intrinsic creative motivation rather than extrinsic motivation or reduced amotivation. Therefore, the findings should be interpreted as preliminary evidence that mindful attention awareness may be linked to academic goal-related functioning mainly through cognitive flexibility and intrinsic creative engagement. Latent profile analysis identified three subgroups of mindful attention awareness: a low mindful attention awareness group, a high mindful attention awareness group, and a moderate mindful attention awareness group. The low mindful attention awareness group exhibited pronounced disadvantages in cognitive flexibility, creative trait motivation, and achievement goal orientation. Overall, this study provides preliminary evidence for the cognitive and motivational processes through which mindful attention awareness is associated with academic goal-related functioning among medical students and supports the development of differentiated mindfulness-informed interventions in medical education.

## Data Availability

The raw data supporting the conclusions of this article will be made available by the corresponding author upon reasonable request, without undue reservation.
